# Ultrafast Superradiant Scintillation from Isolated Weakly Confined Perovskite Nanocrystals

**DOI:** 10.1002/adma.202500846

**Published:** 2025-03-21

**Authors:** Matteo L. Zaffalon, Andrea Fratelli, Zhanzhao Li, Francesco Bruni, Ihor Cherniukh, Francesco Carulli, Francesco Meinardi, Maksym V. Kovalenko, Liberato Manna, Sergio Brovelli

**Affiliations:** ^1^ Dipartimento di Scienza dei Materiali Università degli Studi di Milano Bicocca via R. Cozzi 55 Milano 20125 Italy; ^2^ Istituto Italiano di Tecnologia via Morego Genova 16163 Italy; ^3^ Department of Chemistry and Applied Bioscience ETH Zürich Zürich 8093 Switzerland; ^4^ Laboratory for Thin Films and Photovoltaics and Laboratory for Transport at Nanoscale Interfaces Empa – Swiss Federal Laboratories for Materials Science and Technology Dübendorf 8600 Switzerland

**Keywords:** fast timing, giant oscillator strength, lead halide perovskite nanocrystals, scintillation, superradiance

## Abstract

Efficiency and emission rate are two traditionally conflicting parameters in radiation detection, and achieving their simultaneous maximization can significantly advance ultrafast time‐of‐flight (ToF) technologies. In this study, it is demonstrated that this goal is attainable by harnessing the giant oscillator strength (GOS) inherent to weakly confined perovskite nanocrystals, which enables superradiant scintillation under mildly cryogenic conditions that align seamlessly with ToF technologies. It is shown that the radiative acceleration due to GOS encompasses both single and multiple exciton dynamics arising from ionizing interactions, further enhanced by suppressed non‐radiative losses and Auger recombination at 80 K. The outcome is ultrafast scintillation with 420 ps lifetime and light yield of ≈10 000 photons/MeV for diluted NC solutions, all without non‐radiative losses. Temperature‐dependent light‐guiding experiments on test‐bed nanocomposite scintillators finally indicate that the light‐transport capability remains unaffected by the accumulation of band‐edge oscillator strength due to GOS. These findings suggest a promising pathway toward developing ultrafast nanotechnological scintillators with optimized light output and timing performance.

## Graphical abstract

Efficiency and scintillation velocity are critical for high‐energy and medical physics. These parameters, typically conflicting in conventional scintillators, are simultaneously optimized by exploiting the giant oscillator strength of CsPbBr_3_ nanocrystals, leading to radiatively accelerated (multi)excitonic emission with unity efficiency without detrimental light transport losses in polymeric nanocomposites. This opens up interesting developments in fast‐timing radiation detection based on colloidal nanocrystals.

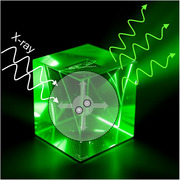

## 1 Introduction

The development of highly efficient and ultrafast scintillators is at the forefront of radiation detection research,^[^ ^1^ ^]^ particularly for high‐precision time‐of‐flight positron emission tomography (ToF‐PET) scanners in oncology,^[^ ^2^ ^]^ and for 4D tracking (*x,y,z,t*) and 5D calorimetry (*x,y,z,t,E*) of nuclear events in high‐brightness hadron colliders.^[^ ^3^ ^]^ Fast scintillation kinetics enable high time resolution and the discrimination of nearby events by reducing pileup. Simultaneously, a high light yield (*LY*), defined as the number of photons emitted per unit of deposited energy, is necessary to ensure a statistically significant event estimate against noise.^[^ ^4^ ^]^ To meet these demands, a scintillator must combine a strong interaction with ionizing radiation, facilitated by a high average atomic number (*Z*_AV_), minimized non‐radiative losses, a large transition oscillator strength, and sufficient radiation stability (also referred to as radiation hardness) to sustain the strong irradiation levels in high energy physics contexts.^[^ ^5^ ^]^ Unfortunately, conventional inorganic or plastic scintillators alone are increasingly inadequate. Inorganic scintillators have slow lifetimes (typically tens of nanoseconds or longer),^[^ ^6^ ^]^ while plastic scintillators have insufficient *Z*_AV_ and low radiation hardness.^[^ ^7^ ^]^ To circumvent these limitations for ToF‐PET, so‐called meta‐scintillator schemes are being explored,^[^ ^8^ ^]^ which leverage energy‐sharing processes from heavy inorganic materials (such as lutetium‐yttrium oxyorthosilicates or bismuth germanates) to molecular emitters with decay times of a few nanoseconds.^[^ ^9^ ^]^

A promising materials‐based approach with potential groundbreaking impact in all applications of ultrafast radiation detection involves the use of colloidal nanocrystals (NCs) of direct bandgap semiconductors,^[^ ^10^ ^]^ particularly lead halide perovskites,^[^ ^11^ ^]^ as nanoscintillators. These NCs offer affordable synthetic scalability and fast emission of molecular dyes combined with the high *Z*_AV_ and radiation stability of inorganic materials,^[^ ^11f^ ^]^ effectively merging the advantages of both existing material systems. Crucially, the quantum confinement regime characteristic of NCs further introduces unique nanoscale physical properties, such as size tunability of the emission wavelength^[^ ^11^, ^12^ ^]^ and large exciton and biexciton binding energies^[^ ^13^ ^]^ that do not occur in bulk or molecular materials, which push the optical and timing capabilities for ToF scintillation to unprecedented levels. A notable example is the recently demonstrated ultrafast scintillation, occurring in hundreds of picoseconds, achieved through the decay of multi‐excitons formed upon the interaction of NCs with ionizing radiation,^[^ ^10^, ^11^, ^14^ ^]^ which significantly surpasses the monomolecular decay observed in organic dyes.

Despite their potential, the study of NCs for ultrafast scintillation is still in its early stages, and powerful photophysical motifs demonstrated under conventional (non‐ionizing) optical pumping have yet to be explored in the context of ionizing excitation. One compelling process that fully radiatively accelerates emission, without activating competing channels, such as concentration quenching or nonradiative Auger recombination – that is the nonradiative annihilation of one exciton in favor of a third carrier,^[^ ^13a^ ^]^ we invite readers to refer to Ref,^[^ ^15^ ^]^ which highlights Lise Meitner's contribution to the discovery of the process commonly known as Auger recombination – that affects the multi‐exciton regime, is the so‐called giant oscillator strength (GOS).^[^ ^16^ ^]^ This results from the coherent coupling of transition dipoles that sustain exciton delocalization over multiple unit cells, which can give rise to collective ultrafast superradiant emission, a phenomenology that has been observed in epitaxial quantum wells,^[^ ^16^, ^17^ ^]^ molecular solids and aggregates^[^ ^18^ ^]^ and in quantum‐confined nanophases in inorganic matrixes.^[^ ^19^ ^]^

**Figure 1:**
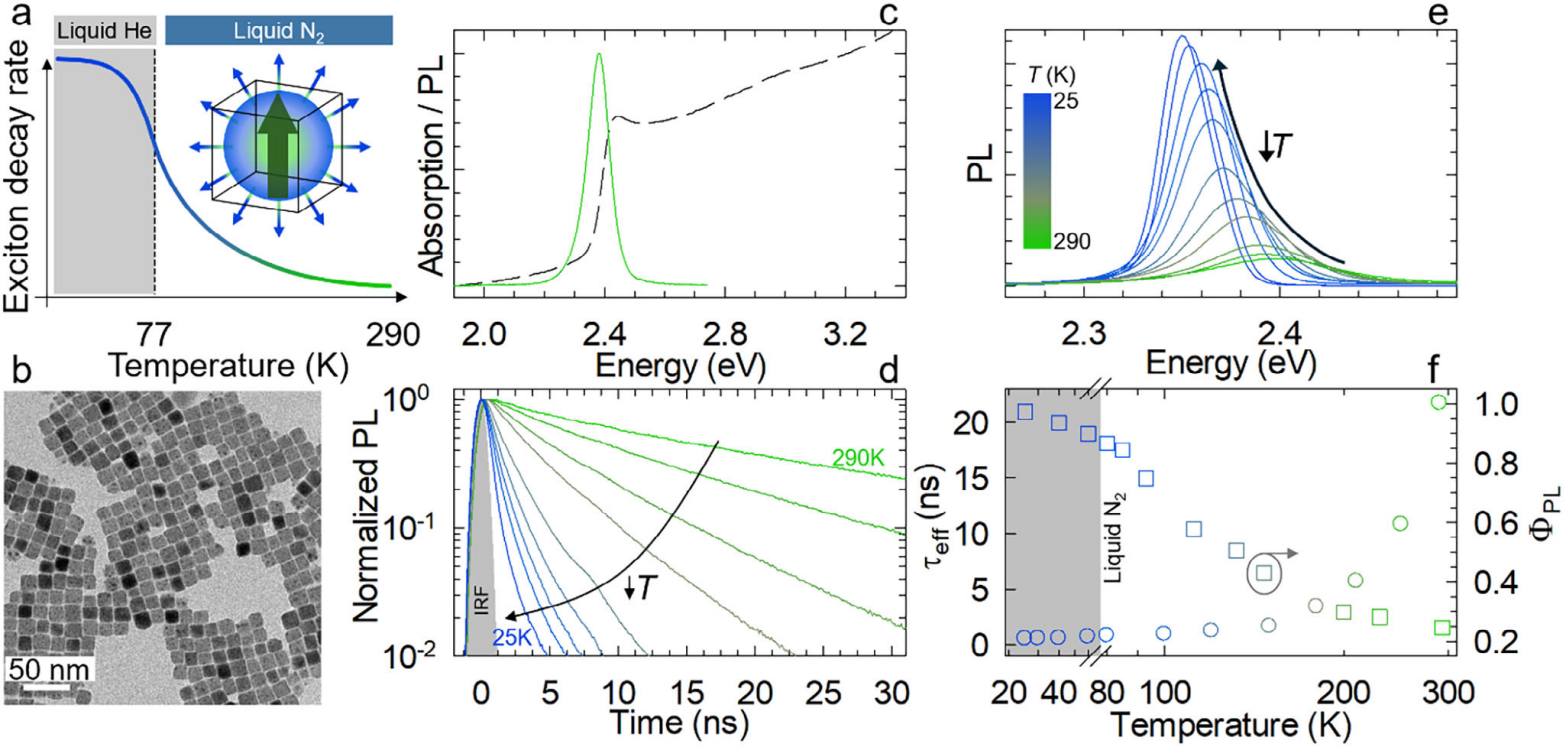
Structural and *T*‐dependent optical properties of single excitons. a) Schematic of the effect of GOS on the decay kinetics in weakly confined CsPbBr_3_ NCs (typically with lateral size ≥9 nm or so) with decreasing temperature. b) High‐resolution TEM micrographs of CsPbBr_3_ NCs. c) Normalized optical absorption (dashed line) and PL (solid line) spectra of CsPbBr_3_ NCs in toluene. d) Normalized *T*‐dependent PL decay traces of CsPbBr_3_ NCs excited using ps‐pulsed 3.06 eV laser operated at 1 MHz. e) *T*‐controlled PL spectra of CsPbBr_3_ NCs in the 290–25 K range when excited at 3.06 eV. f) PL efficiency (*Ф_PL_*) values (squares) obtained rescaling the PL efficiency measured at room temperature for the integrated area of *T*‐dependent PL spectra from “e”. The circles correspond to the measured effective PL decay time from “d”. The same color scheme for temperatures applies throughout the figure.

The formation of giant transition dipoles further underlies the reduction of the gain threshold^[^ ^20^ ^]^ and the radiative acceleration of luminescence in colloidal chalcogenide nanoplatelets^[^ ^10^, ^21^ ^]^ and weakly confined CsPbBr_3_ NCs with decreasing temperature. GOS has recently been shown to produce accelerated emission from both individual particles^[^ ^22^ ^]^ and NC superlattices,^[^ ^23^ ^]^ which is highly promising for quantum light sources.^[^ ^24^ ^]^ Such capability of individual CsPbBr_3_ NCs is enabled by their unique, compared to conventional NCs, prevalence of the radiative recombination through the so‐called bright triplet state at cryogenic temperatures.^[^ ^25^ ^]^ Suppression of thermal quenching and phonon scattering at mild cryogenic temperatures is thus accompanied by the thermalization of excitation in the bright excitonic triplet substate resulting in a massive acceleration of temporal kinetics at near‐unity emission yield (see scheme in **Figure** **1**a). This is invaluable for the design of ultrafast emitters because it breaks the common dichotomy between luminescence efficiency and decay rate, which are typically anticorrelated in conventional scintillators, where shorter emission lifetimes are achieved by controlled activation of additional non‐radiative pathways that quench the efficiency. Also, crucially for scintillation, a large particle size enhances the energy deposition capability of NCs following interaction with ionizing radiation, promoting the formation of fast‐emitting high‐order multiexcitons,^[^ ^26^ ^]^ which are less affected by nonradiative Auger recombination owing to the universal scaling law of the Auger time constant with the particle volume (τ_*AR*_∝*V*).^[^ ^27^ ^]^ Importantly, as we show here, the mid‐range translational order in large NCs also leads to a temperature dependence of the Auger process imposed by the conservation of energy and momentum laws (typical of bulk solids), resulting in the complete suppression of Auger losses at low temperatures. Overall, this makes multi‐exciton scintillation more efficient than in smaller particles even at room temperature, and pushes the multi‐exciton yield to unit values in mild cryogenic conditions due to the complete suppression of Auger losses. The combination of these unique properties of weakly confined CsPbBr_3_ NCs, namely, single particle GOS, high interaction capability with ionizing radiation, and suppressed Auger decay, offers new grounds for the desired simultaneous optimization of timing and efficiency performance in a new class of ultrafast superradiant nanoscintillators, a field that has never been explored.

Here, we demonstrate the potential of GOS in weakly confined CsPbBr_3_ NCs for ultrafast radiation detection by studying the photophysics and scintillation down to liquid nitrogen temperature, a mild cryogenic condition readily accessible for medical instrumentation and typical of many high‐energy physics experiments. Photoluminescence (PL) measurements as a function of temperature on CsPbBr_3_ NCs with lateral size of 15.9 nm confirm the occurrence of single particle GOS, resulting in 20‐fold acceleration of the luminescence lifetime at 80 K accompanied by the saturation of the PL quantum yield (*Φ_PL_*) at near unity values. This behavior is remarkably followed also by biexcitons, probed here via time‐resolved PL and transient absorption (TA) experiments as a function of temperature and excitation fluence, showing a gradual radiative decrease of the biexciton lifetime accompanied by the complete suppression of the nonradiative Auger recombination, leading to accelerated biexciton dynamics with nearly unity efficiency at 80 K. These behaviors are completely preserved under ionizing excitation resulting in fully radiative ultrafast scintillation with 420 ps decay time and *LY* as high as 10 000 photons/MeV at 80 K for a diluted NC solution which is highly promising for ToF radiation detection technologies. Finally, light propagation measurements of the luminescence of testbed nanocomposites containing the same large CsPbBr_3_ NCs, corroborated by Monte Carlo ray tracing simulations, indicate that the temperature‐induced spectral modifications (narrowing and redshift) do not affect light transport, resulting in a net positive enhancement of the scintillation output and preserved ultrafast dynamics. These results, therefore, suggest a potential strategy for the future development of high‐performance nanotechnological scintillators for ultrafast radiation detection.

## 2 Results and Discussion

CsPbBr_3_ NCs were synthesized by a modified hot injection procedure described in the Methods^[^ ^28^ ^]^ followed by ligand exchange with didodecyldimethylammonium bromide. Structural analysis by high‐resolution transmission electron microscopy (HR‐TEM) and X‐ray diffraction (XRD) in Figure 1b and Figure S1 (Supporting Information) shows that the NCs have a cuboid shape with an average size of 15.9 ± 1.4 nm, well above the Bohr diameter (≈7 nm) of CsPbBr_3_,^[^ ^12b^ ^]^ and orthorhombic crystal structure. In order to investigate the optical and scintillation properties of isolated NCs without additional effects due to interparticle interactions and/or mutual cross‐excitation effects via released photoelectrons, the NCs were dispersed in a non‐scintillating solvent (octane) at relatively low concentration (0.7 wt.%) and tested in this form, except where explicitly noted. The room temperature optical absorption spectrum and PL profiles show an absorption edge at ≈2.42 eV and PL peak at 2.38 eV (Figure 1c), close to the emission energy of bulk CsPbBr_3_ (≈2.38 eV).^[^ ^29^ ^]^ The room temperature PL dynamics (Figure 1d) measured at vanishingly low excitation fluence to ensure the production of only single excitons (average NC exciton occupancy 〈N〉 ≈0.05) is essentially single exponential with an effective lifetime (defined as the time after which the signal has dropped by a factor equal to *e*) of $\tau_{X}^{PL}\left(300K\right)$ ≈21 ns (corresponding to a single exciton decay rate, *k_X_* (300K) = 48 MHz), measurably longer than smaller CsPbBr_3_ NCs due to the stronger *s‐p* hybridization in larger particles.^[^ ^30^ ^]^ Note that we consider the single exciton lifetime as the radiative lifetime, since non‐radiative losses in NCs are typically due to ultrafast trapping processes that occur prior to exciton recombination.

To promote the formation of GOS, we cooled down the NC solution and collected both the steady‐state and time‐resolved PL still carefully adjusting the excitation fluence to operate in the single exciton regime. The PL spectra in the 290–25 K temperature range are reported in Figure 1e showing an almost 4‐fold intensification upon cooling together with the spectral narrowing and red‐shifting characteristic of perovskite NCs due to lattice expansion and reduced phonon coupling.^[^ ^31^ ^]^ As a result, the corresponding PL efficiency in Figure 1f increases from *Φ_PL_* ≈21% (as measured with an integrating sphere) at room temperature up to nearly 90% at 80 K due to suppression of trapping (extracted from the raise of integrated PL intensity versus *T*). In contrast to conventional observations of slower decay dynamics at low temperatures and in agreement with previous reports,^[^ ^22^, ^32^ ^]^ cooling the NCs led to over twenty‐fold acceleration of the single exciton lifetime to $\tau_{X}^{PL}\left(80K\right)$ = 830 ps (Figure 1d,f). Notably, the PL efficiency is essentially maximized at liquid N_2_ temperature, with marginal, <10%, improvement below 30 K when the *Φ_PL_* reached ≈97%, which corroborates the technological validity of the approach.

**Figure 2:**
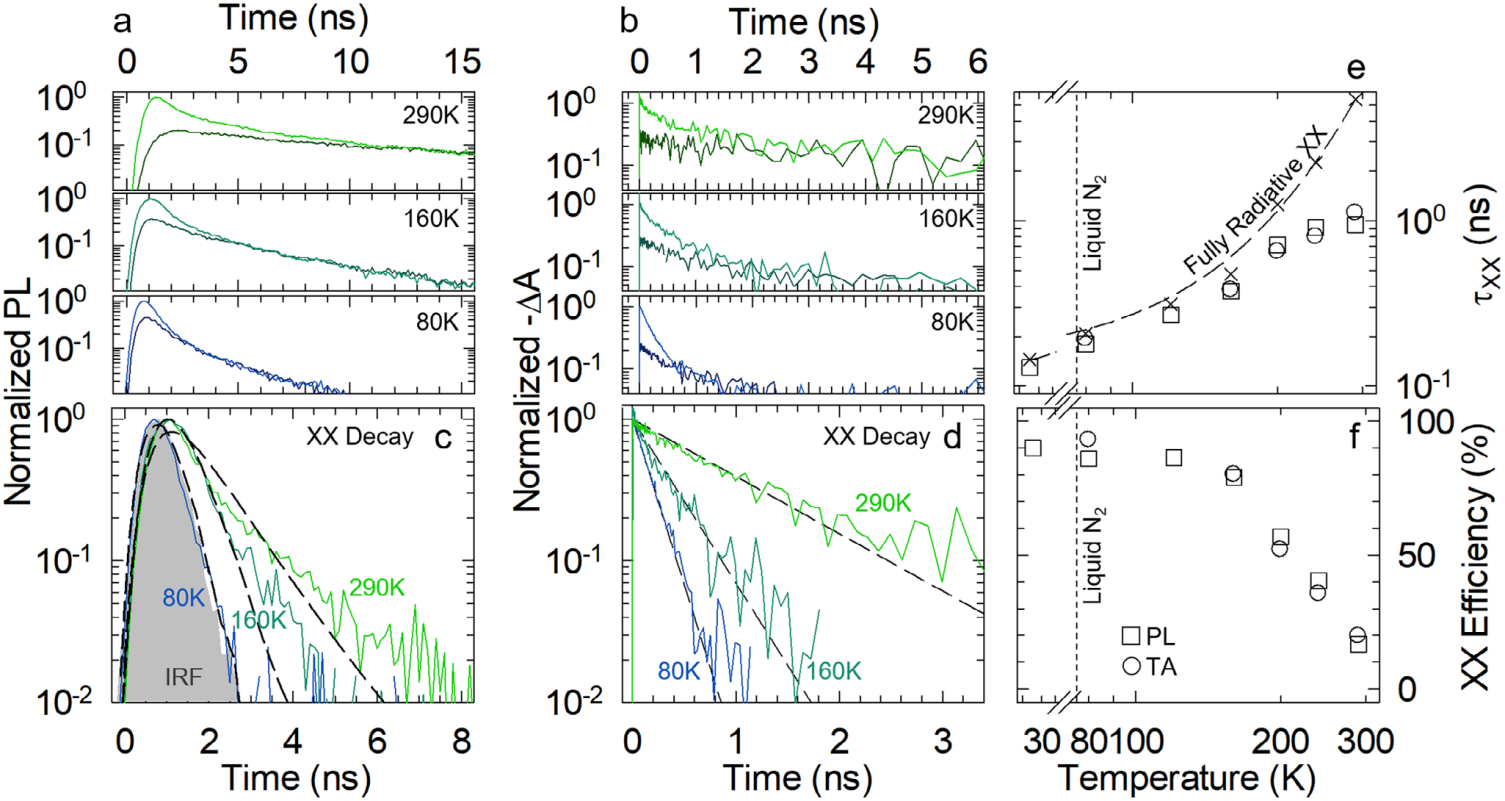
*T*‐dependent biexciton efficiency and Auger processes. a) PL decay traces of CsPbBr_3_ NCs collected at controlled temperatures (290, 160, and 80 K) excited with pulsed 3.06 eV fs‐laser at low (dark colored) and high (light colored) fluences. b) Transient absorption bleach recovery dynamics collected at the same temperatures in “a” under similar low‐ and high‐fluence regimes. c) PL decay traces of the biexcitonic components extracted from “a”. The grey shaded area represents the instrument response (IRF), and the dashed lines correspond to the best fit with a single‐exponential decay function convoluted with the IRF. d) Differential TA curves of the biexciton components extracted from “b” together with the best single exponential decay fit (dashed lines). e) Temperature dependence of the biexciton lifetime (τ_*XX*_) extracted from fluence‐dependent PL (squares) and TA (circles) measurements. The crosses correspond to the theoretical fully radiative biexciton lifetime calculated as τ_*x*_(*T*)/4. The dashed line is a guide for the eye. f) Calculated biexciton efficiency as a function of temperature from both set of PL and TA measurements. The same color scheme applies to the figure.

Having confirmed the necessary photophysical prerequisites of weakly confined CsPbBr_3_ NCs in the single‐excitonic regime, before moving to the study of scintillation, we assessed the effect of temperature on the multi‐excitonic recombination that underpins the scintillation process. To this end, we performed PL and TA measurements as a function of temperature with increasing excitation fluence. **Figure** **2**a,b shows the low and high fluence PL and TA bleaching dynamics, respectively, at three representative temperatures. Consistent with the PL data in Figure 1d, in both experiments, lowering the temperature accelerates single exciton decay while increasing the excitation fluence leads to the rise of a fast component due to biexciton recombination. Following the procedure introduced by Klimov^[^ ^33^ ^]^ for the analysis of multi‐exciton dynamics, Figure 2c,d shows the biexciton decay curves extracted by subtracting the single‐exciton dynamics from the respective high fluence curve after tail normalization at long delay times. In agreement with previous reports,^[^ ^34^ ^]^ both experiments yield a room temperature biexciton lifetime τ_*XX*_(300*K*) ≈1 ns (corresponding to a biexciton rate *k_XX_* (300*K*) =  1 ns^−1^). Considering the statistical link between the single and biexciton radiative rates ($k_{XX}^{rad}$ = 4*k_X_* ), this corresponds to a biexciton efficiency $\Phi_{XX}\left(300K\right)=k_{XX}^{rad}\;\left(300K\right)/k_{XX}\left(300K\right)$ = 19%. We emphasize that because of the temperature dependence of both GOS and the population equilibrium between the excitonic substates, and the Auger recombination in weakly confined NCs, all decay rates are functions of temperature. This is not the case in strongly confined particles where the Auger recombination is temperature independent due to the breakdown on momentum conservation requirement.^[^ ^27^, ^35^ ^]^ As a result, following the decrease of τ_*X*_(*T*) with decreasing temperature, the biexciton decay also gradually accelerates, as further quantified by the corresponding experimental τ_*XX*_(*T*) in Figure 2e, which approaches the fully radiative value at ≈100 K, indicating essentially complete suppression of Auger recombination. This important effect of temperature on biexciton photophysics is further highlighted in Figure 2f, where we report the Φ_*XX*_ versus *T* trend showing saturation at >90% at liquid N_2_ temperatures. Crucially, similar to the single‐exciton behavior discussed in Figure 1, this increase in biexcitonic recombination efficiency is accompanied by its full radiative acceleration, effectively extending the efficiency‐velocity paradigm to multi‐carrier dynamics. We note that the time dynamics of biexcitons as a function of temperature in Figure 2 suggests that biexcitons in our NCs do not experience any measurable additional GOS over their respective single excitons. This differs from the observation of biexciton GOS in bulk halides at low temperatures,^[^ ^36^ ^]^ which exceeds the oscillator strength of single excitons by orders of magnitude due to the much larger size of biexcitons, effectively involving a massively larger number of unit cells, and is likely due to the size limit imposed on both single and biexcitons by quantum confinement.

**Figure 3:**
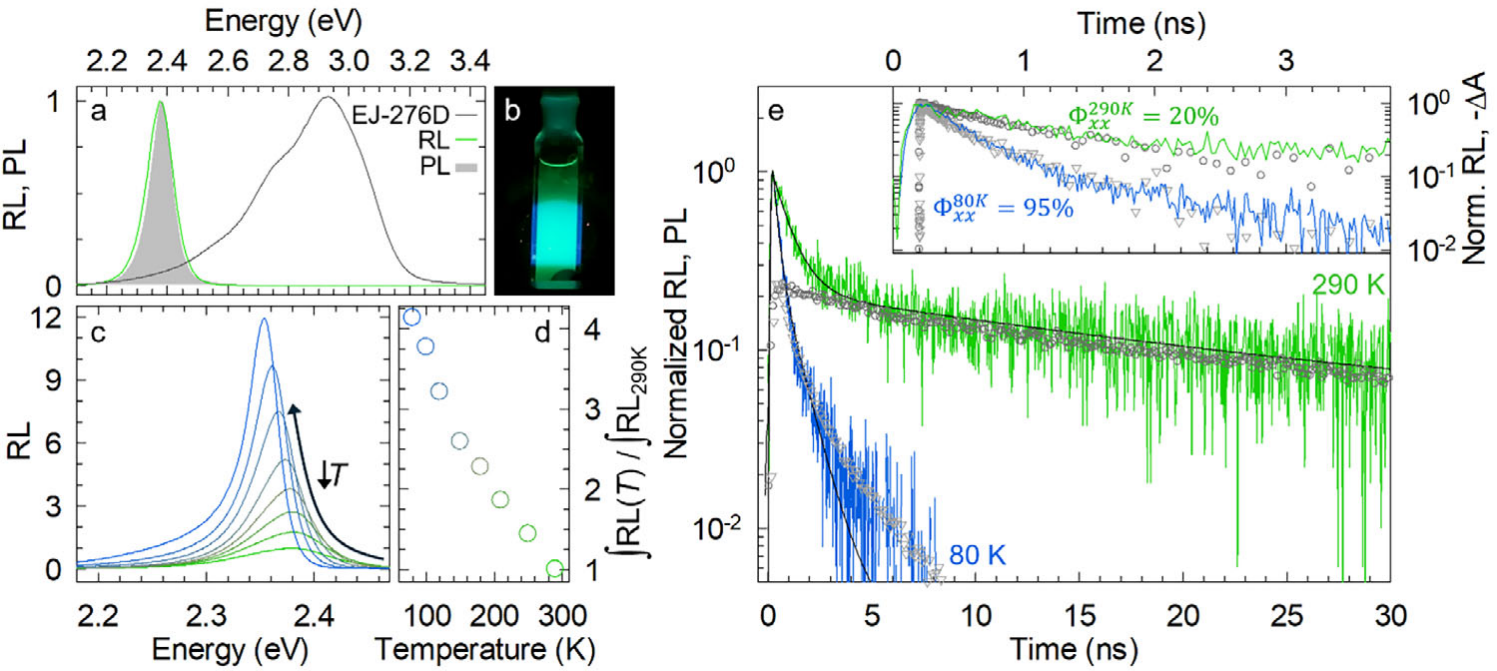
GOS‐enhanced scintillation of CsPbBr_3_ NCs. a) RL spectrum of a solution of CsPbBr_3_ NCs dispersed in octane (0.7 wt%) together with a commercial EJ‐276D plastic scintillator used as reference. The corresponding normalized PL spectrum of CsPbBr_3_ NCs is shown as a shaded grey area. b) Picture of an octane colloidal suspension of CsPbBr_3_ NCs under X‐ray excitation. c) X‐ray excited (≈7 keV) RL spectra of CsPbBr_3_ NCs as a function of temperature in the range 290–80 K. d) Temperature dependence of the spectrally integrated RL intensity extracted from “c”. The values have been normalized for the value at 290 K. e) Time‐resolved PL (symbols) and RL (colored lines) decay traces of CsPbBr_3_ NCs normalized over the single‐excitonic tail, both at 290 K (circles, green line) and at 80 K (triangles, blue line). The solid black lines are the best fit of the scintillation decays (see Supporting Information for details). Inset: comparison of the scintillation dynamics and the bleach recovery dynamics of biexciton components (symbols) extracted from TA measurements at 290 and 80 K. The same color scheme applies to the inset.

Based on the promising optical properties of our CsPbBr_3_ NCs in both the single and multi‐exciton regime, we proceeded to demonstrate their potential in scintillation using X‐ray excitation. The RL spectrum of a colloidal suspensions of NCs in octane (0.7 wt%) is shown in **Figure** **3**a and closely matches its corresponding PL profile indicating that the RL originates from the same band‐edge exciton states. A photograph of the sample under X‐ray irradiation in shown in Figure 3b. The *LY* of 150 µL (in a 0.5 cm high cylindrical cuvette) of the colloidal suspension measured in the same excitation and collection geometry at room temperature as a commercial plastic scintillator EJ‐276D (*LY* = 8600 photons/MeV) of the same volume (in both cases resulting in essentially complete deposition of the incident X‐ray excitation) is 2400 ± 100 photons/MeV, consistent with previous results on diluted CsPbBr_3_ NCs composites.^[^ ^11k^ ^]^ We emphasize that the definition of *LY* for colloidal NCs is still a matter of debate as it depends on both single particle effects (i.e., stopping power, exciton and biexciton emission yields) and extensive phenomena such as outcoupling and interparticle cross‐excitation by photoelectrons released outside a single NC following primary excitation events, which can be significantly improved by increasing the particle concentration and packing, as recently demonstrated using dense CsPbBr_3_ NCs‐based composites.^[^ ^11^, ^37^ ^]^ Since the scope of this work is to demonstrate the potential of intraparticle GOS, the sample concentration was specifically chosen to avoid interparticle effects. Consistent with the PL trend, the RL peak at 2.38 eV red‐shifts, narrows, and intensifies by over four‐fold upon cooling (Figure 3c,d), reaching a *LY*(80K) ≈10 000 photons/MeV, corresponding to the radiative limit of the scintillation efficiency for this system.

Also importantly for ToF applications, the RL kinetics undergoes a similar acceleration as the respective PL. Specifically, the room temperature RL decay trace (Figure 3e) features two contributions typical of CsPbBr_3_ NCs:^[^ ^11^, ^14^ ^]^ a main component with relative weight *w*_X_(300K) = 80% and lifetime $\tau_{RL}^{X}\left(300K\right)$ = 20 ns which coincides with the low‐fluence PL decay (circles in Figure 3e) and is attributed to the RL of single excitons, and a faster contribution with *w_XX_* (300K) = 20% and lifetime $\tau_{RL}^{XX}\left(300K\right)$ = 850 ps closely matching the corresponding biexciton decay extracted from the TA dynamics (symbols in the inset of Figure 3e) and thus attributed to biexciton emission with efficiency of ≈20%. Consistent with recent results, the ratio between the amplitudes of the *X* and *XX* components, following the typical approach for ultrafast kinetic studies in NCs, yields an estimated excitonic population 〈*N*〉 =  3.5 as expected for ≈16 nm NCs.^[^ ^33^, ^34^ ^]^

At 80 K, both the single and biexciton decays are significantly accelerated with $\tau_{RL}^{X}\left(80K\right)$ = 920 ps and $\tau_{RL}^{XX}\left(80K\right)$ = 220 ps, yielding a biexciton efficiency as high as 95% also under X‐ray excitation. As a result of the faster dynamics in both excitonic regimes, the so‐called effective scintillation lifetime, calculated as the weighted harmonic average of the single and biexciton and contributions (${\left(\tau_{RL}^{EFF}\right)}^{-1}=\sum \frac{w_{i}}{\tau_{i\;}}),\;$ is as fast as $\tau_{RL}^{EFF}\;\left(80K\right)=$ 420 ps, which is very promising for fast‐timing technologies. The potential advantage of the peculiar fully‐radiative ultrafast photophysics of weakly confined CsPbBr_3_ NCs becomes particularly evident by estimating the coincidence time resolution^[^ ^14b^ ^]^ (*CTR*) ideally achievable in ToF‐PET scanners at mild cryogenic temperatures. This empirical parameter expresses the time required to obtain a statistically relevant signal given the effective time and the *LY* and, although not formalized identically, is also a representative figure of merit for application in ultrafast calorimeters for high luminosity colliders, which require double‐impulse separations of a few nanoseconds in addition to time resolutions of a few tens of ps or less. Using the expression $CTR=3.33\sqrt{\frac{\tau_{RISE}\times \tau_{RL}^{EFF}\left(80K\right)}{\Gamma (80K)}},$ where τ_*RISE*_ is the 10–90% signal rise time here set to 90 ps and Γ(80K)  = 5100 is the estimated number of scintillation photons emitted at 80 K under the characteristic 511 keV gamma excitation used in ToF‐PET, we obtain an estimated *CTR*(80K) = 9 ps, which is very promising for meeting the so‐called 10 ps challenge^[^ ^38^ ^]^ which would allow for improved detection sensitivity along the response line, enabling millimeter spatial resolution in ToF‐PET diagnostics.^[^ ^39^ ^]^ We note that since this study focuses on the potential of collective phenomena to simultaneously enhance both LY and timing capability, the *CTR* serves as a figure of merit that captures both benefits rather than a practical performance indicator for a real PET detector. In a fully developed scintillating device, density and geometry would be optimized to achieve the required stopping power and to maximize light outcoupling to meet operational and spectroscopic requirements at higher γ‐ray energies.

**Figure 4:**
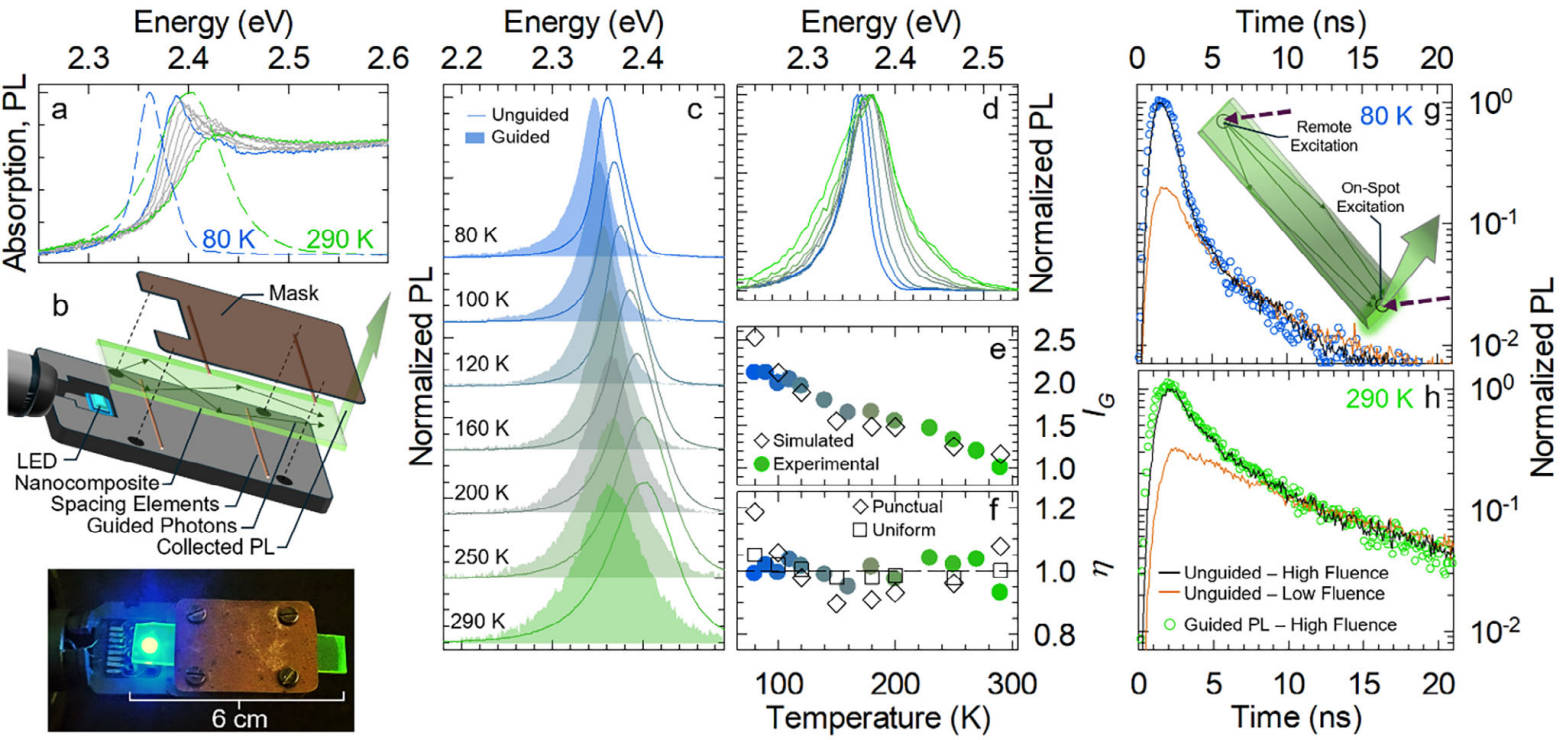
Temperature effects on light transport. a) Optical absorption spectra at decreasing temperature from 290 K (green line) to 80 K (blue line). The corresponding normalized PL spectra at the highest and lowest temperatures are shown with dashed lines. b) Schematic representation and photograph of the experimental configuration used to collect PL spectra shown in “c”. c) Normalized PL spectra (solid lines) at controlled temperatures together with the corresponding guided PL spectra (shaded areas) excited at ≈6 cm from the collection edge. d) Simulated guided PL spectra as a function of temperature. The color scheme of “c” applies. e) Spectrally integrated intensity of the guided PL as a function of temperature. Data were normalized to the value at 290 K. Circles and diamonds represent the experimental and the simulated data, respectively. f) Guided PL emission efficiency (η) as a function of temperature (filled circles for the experimental data) together with corresponding simulated values (empty diamonds). The simulated η‐values under uniform illumination of the waveguide are shown as empty squares. Tail normalized guided (circles) and unguided (lines) PL decay traces at g) 80 K or h) 290 K under low (orange line) and high (black line) excitation fluences.

Finally, given the spectral change in absorption and emission profiles at cryogenic temperatures (**Figure** **4**a) associated with the decrease in thermal disorder and the consequent accumulation of oscillator strength in band‐edge states, it is instructive to evaluate the effect of temperature on the light guiding ability of scintillating waveguides based on weakly confined NCs. This is because, while lowering the temperature reduces the Stokes shift between emission and absorption,^[^ ^40^ ^]^ suggesting more reabsorption losses, the concomitant suppression of homogeneous broadening might reduce the spectral overlap sufficiently to lead to a net improvement in light‐guiding ability. To assess this aspect, we fabricated testbed nanocomposite waveguides (6×0.5×0.1 cm) by optical polymerization of poly‐lauryl methacrylate containing NCs and evaluated the effect of temperature on light propagation according to the experimental scheme shown in Figure 4b. The polymer matrix was chosen to be non‐scintillating in order to study the response of the NCs alone, and the loading was set at 0.1 wt% to keep light scattering low despite the large particle size and the absence of resurfacing steps with compatibilizing ligands. First, the nanocomposites were optically characterized to ensure the preservation of the optical properties of the NCs after radical polymerization.

The PL spectrum and temporal dynamics are shown in Figure S2 (Supporting Information) and demonstrate the substantial stability of the particles with respect to the fabrication process, with a minimal *Φ_PL_* loss of ≈5%. The nanocomposites were then placed in the variable temperature insert of a liquid helium cryostat, which allows the sample to be uniformly cooled without thermal contact with the substrate, thus preserving the waveguiding capability. The experiment then consisted of exciting one end of the nanocomposite with a 465 nm diode (inserted directly into the cryostat, see Figure 4b) and measuring the luminescence emitted from the opposite end (guided, *G*) as a function of temperature. This configuration represents the most unfavorable situation, as the generated light must travel the entire length of the sample before being outcoupled and was specifically chosen to make the effect of temperature on reabsorption more apparent. The luminescence at the point of excitation (unguided, *UG*) was also collected to directly estimate the evolution of the spectral shape and intensity with temperature, which were then used to model the light transport via Monte Carlo simulations. Figure 4c shows the normalized spectra of guided and unguided PL for temperatures ranging from 300 to 80 K, the corresponding simulated guided spectra are shown in Figure 4d. A systematic redshift of the guided spectrum with respect to the corresponding unguided emission is observed due to partial reabsorption and re‐emission by the NCs. Importantly, the intensity of guided luminescence (*I_G_*) gradually increases with decreasing temperature (Figure 4e). To disentangle the effects of light transport and increasing *Φ_PL_* with decreasing temperature, we therefore calculated the propagated emission efficiency, *η = I_G_/I_UG_*, expressed by the ratio between the integrated intensity of the guided and unguided light. As shown in Figure 4f (filled symbols), *η* is found to be temperature‐independent, indicating that the activation of GOS does not come at the expense of the optical performance of the composite. Ray tracing Monte Carlo simulations of light propagation performed using the experimental sample geometry and optical parameters (absorption and PL spectra, *Φ_PL_*) show very good agreement with the experimental data (empty diamonds in Figure 4f). Also importantly, similar side‐by‐side PL measurements performed using pulsed UV laser excitation show that the guided PL retains the same ultrafast multiexciton emission dynamics as in unguided mode both at room temperature and at 80 K (Figure 4g). Finally, the agreement between the experimental and theoretical results further allows us to use the Monte Carlo simulation to model the expected efficiency evolution with temperature under homogeneous illumination of the waveguide body (empty squares, Figure 4f), which might be a condition closer to the real use of scintillator materials in radiation detection (especially in sampling calorimeters). As highlighted by the dashed line in Figure 3f this condition indeed leads to completely temperature‐independent light guiding.

## 3 Conclusion

In conclusion, we have shown that weakly confined CsPbBr_3_ NCs exhibit single particle GOS even under ionizing excitation, resulting in fully radiatively accelerated single and biexciton dynamics at mild cryogenic temperatures. This is accompanied by enhanced single and biexciton emission due to suppressed thermal losses and non‐radiative Auger recombination, effectively allowing the radiative limit of both kinetics and efficiency to be reached without sacrificing light transport capability. Furthermore, we anticipate that margins for further improvement are in principle possible by exploiting the ability of perovskite nanocrystals to self‐assemble into dense superlattices,^[^ ^41^ ^]^ which exhibit further superradiance at the supramolecular level^[^ ^23b,c,f,g^ ^]^ and realistically offer the possibility of increased energy capture upon ionizing excitation and consequently even higher light yields. Finally, their solution processability makes them fully compatible with metascintillator and/or cavity‐based designs, which further enhances the timing capability by increasing the LY and accelerating the radiation decay via the Purcell effect.^[^ ^42^ ^]^ The superradiant scintillation motif thus opens up interesting possibilities for the future development of ultrafast nanotechnological scintillators for fast time‐of‐flight radiation detection.

## Conflict of Interest

The authors declare no conflict of interest.

## Supporting information



Supporting Information

## Data Availability

The data that support the findings of this study are available from the corresponding author upon reasonable request.
