# Supplementary material for: Ultrafast Superradiant Scintillation from Isolated Weakly Confined Perovskite Nanocrystals
Source: Adv Mater. 2025 Mar 21;37(18):2500846. doi: 10.1002/adma.202500846 (PMC12051787; doi:10.1002/adma.202500846)
Supplement: Supplementary file 1 — Supporting Information [file ADMA-37-2500846-s001.pdf]

ADVANCED MATERIALS

Supporting Information

for *Adv. Mater.*, DOI 10.1002/adma.202500846

Ultrafast Superradiant Scintillation from Isolated Weakly Confined Perovskite Nanocrystals

*Matteo L. Zaffalon, Andrea Fratelli, Zhanzhao Li, Francesco Bruni, Ihor Cherniukh, Francesco Carulli, Francesco Meinardi, Maksym V. Kovalenko, Liberato Manna and Sergio Brovelli**

Supporting Information

Ultrafast Superradiant Scintillation from Isolated Weakly Confined Perovskite Nanocrystals

Matteo L. Zaffalon[1], Andrea Fratelli[1], Zhanzhao Li[2], Francesco Bruni[1], Ihor Cherniukh[3], Francesco Carulli[1], Francesco Meinardi[1], Maksym V. Kovalenko[3], Liberato Manna[2], Sergio Brovelli[1*]

[1]*Dipartimento di Scienza dei Materiali, Università degli Studi di Milano Bicocca, via R. Cozzi 55, Milano, Italy*
[2] *Istituto Italiano di Tecnologia, via Morego, Genova, Italy*
[3] *Department of Chemistry and Applied Bioscience, ETH Zürich, Zürich, Switzerland.*
Laboratory for Thin Films and Photovoltaics and Laboratory for Transport at Nanoscale Interfaces, Empa – Swiss Federal Laboratories for Materials Science and Technology, Dübendorf, Switzerland.

Materials and Methods

Materials

Octadecene (90%), cesium(I) carbonate (Cs_2CO_3, 98%), lead (II) acetate trihydrate ($Pb(OAc)_2 \cdot 3H_2O$, 99.99%), oleic acid (OA, 90%), didodecyldimethylammonium bromide (DDABr, 98%), toluene (anhydrous, 99.8%), methyl acetate (99.5%), lauryl methacrylate (LMA, 98%), ethylene glycol dimethacrylate (EGDM, 97.5%), 2,2-dimethoxy-2-phenylacetophenone (Irgacure 651, 99%) were purchased from Sigma-Aldrich. Didodecylamine (> 97%) and Benzoyl bromide (98%) were purchased from Tokyo Chemical Industry (TCI). All chemicals were used without any further purification.

Synthesis of $CsPbBr_3$ nanocubes

$CsPbBr_3$ NCs are synthesized following a previously reported method[1] with minor adjustments. 0.05 mmol of $Cs_2(CO_3)$, 0.2 mmol of $Pb(OAc)_2 \cdot 3H_2O$ and 1.25 mmol of didodecylamine were dissolved in 10 ml of octadecene and 1.5 ml of oleic acid in a 25 ml three-necked flask. The resulting mixture was pumped to vacuum at room temperature for 30 min and then at 100 °C for 1 hour. Then the mixture was changed to a nitrogen atmosphere, and the temperature was raised to 160°C. A benzoyl bromide solution (prepared by mixing 50 μL of benzoyl bromide (0.42 mmol) in 500 μL of degassed octadecene) was swiftly injected, and the reaction was run 2 minutes, after which the flask was swiftly cooled to room temperature by immersion in an ice-water bath. The NCs were precipitated by the addition of a mixture of methyl acetate and toluene (volume ratio of 2:1) to the crude solution, followed by centrifugation at 6000 rpm for 10 min. The precipitate was redispersed in 4 ml of anhydrous toluene.

Ligand Exchange with DDABr

2 mL of DDABr solution (0.2M in toluene) was added to the 4 mL of $CsPbBr_3$ NCs solution under vigorous stirring for 1 minute. Then, the NCs were washed by addition of 4 mL of methyl acetate followed by centrifugation at 6000 rpm for 10 minutes. The precipitate was redispersed in 4 mL toluene. One additional cycle of ligand exchange was carried out following the same procedure. Finally, the NCs solution was washed by the addition of 4 mL of methyl acetate followed by centrifugation and resuspension in toluene. This final washing was done twice. The final NCs was redispersed in 4 mL toluene for XRD and TEM characterizations. We noted that, without this final washing procedure, the $CsPbBr_3$ NCs treated with DDABr tend to degrade over time.

Structural characterization

X-ray Diffraction (XRD). XRD analysis was performed on a PANanalytical Empyrean X-ray diffractometer equipped with a 1.8 kW Cu Kα ceramic X-ray tube (λ = 1.5406 Å) and an operating at 45 kV and 40 mA. $CsPbBr_3$ NC solutions were first concentrated under a flow of nitrogen, then they were drop-cast on a zero-diffraction single crystal substrate.

Transmission Electron Microscopy (TEM) Characterization. Bright-field TEM (BF-TEM) images with a large field of view were acquired on a JEOL JEM-1400Plus microscope with a thermionic gun (LaB_6 crystal), operated at an acceleration voltage of 120 kV.

Fabrication of polymer nanocomposite

The polymerization was performed by adding 2,2-dimethoxy-2-phenylacetophenone photo-initiator (0.33 wt%) to a colloidal suspension of NCs in LMA/EGDM (80:20). The mixture was then transferred into a sealed mold consisting of two plain glass slabs separated by a silicone gasket and placed in a polymerization chamber with continuous 365 nm light exposure. After 15 minutes of UV irradiation, an optical-grade nanocomposite without macroscopic phase segregation was obtained.

Optical characterization

The UV-Vis absorption spectra, also measured as a function of temperature, were collected using a Lambda 950 spectrophotometer (Perkin Elmer) equipped with an integrating sphere. Photoluminescence (PL) spectra and corresponding PL quantum yield were measured under low-intensity continuous-wave (*cw*) excitation at 3.06 eV (405 nm), collecting the emitted light with an integrating sphere and a high-resolution CCD spectrometer (TM series, Hamamatsu). PL decay dynamics were recorded using a Si-

phototube coupled to a Cornerstone 260 1/4 m VIS-NIR monochromator (ORIEL) and a time-correlated single-photon counting unit (TCSPC, time resolution 100 ps), while the sample was excited at 3.06 eV using a ~70-ps pulsed laser. Temperature-dependent measurements were carried out by mounting the sample in a closed-circuit He cryostat with optical access. Ultrafast transient absorption (TA) spectroscopy measurements were conducted using a Helios TA spectrometer (Ultrafast Systems). The laser source was a 10 W Hyperion amplified laser, operated at 1.875 kHz and producing ~260 fs pulses at 1030 nm. This was coupled to an independently tunable APOLLO-Y optical parametric amplifier (OPA) from the same supplier, producing excitation pulses at 3.1 eV synchronously chopped at 937 Hz. The probe beam was a white-light supercontinuum. The PL dynamics in the low- and high-fluence regimes were collected using the previously described TCSPC setup, coupled to the fs-pulsed output of the OPA to achieve high fluences. All spectroscopic and radiometric measurements were performed on colloidal dispersions of NCs in octane, a solvent chosen specifically for its lack of intrinsic scintillation to ensure that the radioluminescence signal originated solely from the NCs. Temperature-dependent light transport measurements were performed on a 0.1 wt% loaded PLMA nanocomposite placed in a liquid-He closed-circuit cryostat operated in an evaporated He atmosphere at ~10 mbar, ensuring uniform cooling with minimal contact with the sample holder to preserve the light-guiding properties. In this configuration, PL was excited using a narrow emitting 465 nm diode (VLDB1232G-08, Vishay) operated at constant current using a Keithley Model 2450 SourceMeter.

RL measurements

Unfiltered X-rays were produced using a Philips PW2274 X-ray tube with a tungsten target, equipped with a beryllium window and operated at 20 kV to produce a continuous X-ray spectrum through bremsstrahlung. Cryogenic RL measurements were conducted in the temperature range of 20−290 K using a closed-cycle He cryostat. The scintillation light was detected using a liquid-nitrogen-cooled, back-illuminated, UV-enhanced CCD detector (Jobin Yvon Symphony II), coupled to a monochromator (Jobin Yvon Triax 180) with a 100 lines/mm grating.

LY measurements

Light yield values were determined by comparing the integrated RL intensity under 20 kV X-ray excitation (⟨E⟩ ~ 7 keV) with identical experimental conditions for a 0.7 wt% octane solution of $CsPbBr_3$ NCs placed in a 5 mm long crucible and a commercial EJ-276D plastic scintillator (LY = 8600

photons/MeV) of the same size and geometry used as a reference. In both cases, the sample size was chosen to ensure complete attenuation of the excitation beam.

Time resolved scintillation measurements

The time-resolved RL was measured using a pulsed X-ray source consisting of a 405 nm ~70-ps pulsed laser hitting the photocathode of an X-ray tube (N5084, Hamamatsu) set at 40 kV. The emitted scintillation light was collected using an FLS980 spectrometer (Edinburgh Instruments) coupled to a PicoHarp 300 hybrid photomultiplier tube operating in TCSPC mode. RL dynamics at low temperatures were collected in the same setup while keeping the sample constantly submerged in liquid nitrogen. The RL decay curves were analysed using a least-squares fitting approach with the following formula, which accounts for the convolution with the instrument response function (IRF):

$$F(t) = IRF(t) \otimes \left(H(t - t_0) \cdot \left[\sum_{i=1}^{2} a_i \cdot e^{-t/\tau_i} \right] \right) + C$$

where t_0 corresponds to the start of the emission process, *C* is the electronic background noise floor, and *H* is the Heaviside function. The experimental IRF was well described by a Gaussian profile (FWHM = 120 ps), and the weight of each component (w_i) was calculated as the integral of each convoluted function over the entire time window. The average lifetime was calculated using the re-normalized ratio of all components τ_i according to:

$$\tau_{eff} = \left(\frac{\tau_1}{w_{1n}} + \frac{\tau_2}{w_{2n}} \right)^{-1}, \qquad w_{in} = \frac{w_i}{w_1 + w_2}$$

The same model was used to fit the biexciton dynamics obtained from the time-resolved PL measurements in Figure 2.

Monte Carlo Ray-Tracing Simulation.

Simulations of waveguiding performance were carried out using a Monte Carlo ray-tracing method, where photon propagation follows the laws of geometrical optics. Because the plastic scintillator thickness is much larger than the light coherence length, interference effects were neglected. The stochastic nature of the simulations was reflected by not splitting rays at interfaces, but instead treating them as either transmitted or reflected, with probabilities proportional to energy fluxes given by Fresnel's laws. The dependence of these probabilities on the polarization state of the incident ray (e.g., s or p

polarization) was also considered. Within the nanocomposite, the inverse transform sampling method was used to generate the optical path length before absorption by the NCs, following an exponential attenuation law determined by the wavelength-dependent absorption cross section, $\sigma(\lambda)$, and the NC concentration, $N(\lambda)$, yielding the attenuation coefficient, $k(\lambda) = \sigma(\lambda)N(\lambda)$. Since the mean path length (inverse of the attenuation coefficient) was always much greater than the average distance between NCs, there was no need to track individual NCs, allowing the nanocomposite (PLMA + NCs) to be treated as a uniform medium. Once absorbed by an NC, the photon's fate – either reemission or nonradiative relaxation – was determined by Monte Carlo sampling based on the experimental emission quantum yield. The direction of reemission was distributed uniformly, and the reemission wavelength was determined using rejection sampling based on the experimentally obtained NC luminescence spectrum. The final fate of each photon was either loss due to nonradiative recombination or escape from the nanocomposite via one of its interfaces. Each simulation typically consisted of 10^5 - 10^7 repetitions to achieve adequate statistical averaging. This approach allowed for the evaluation of various observables and the addition of additional processes. The nanocomposite was modelled as a rectangular parallelepiped with dimensions 6.0×0.5×0.1 cm. The experimental illumination condition was modelled by positioning the primary photon origin close to one end of the composite (along the longer axis), while uniform illumination was achieved by randomly generating primary photons within the entire nanocomposite volume. Simulated guided PL spectra and corresponding intensities were reconstructed from the photons escaping from the opposite 0.5×0.1 cm face.

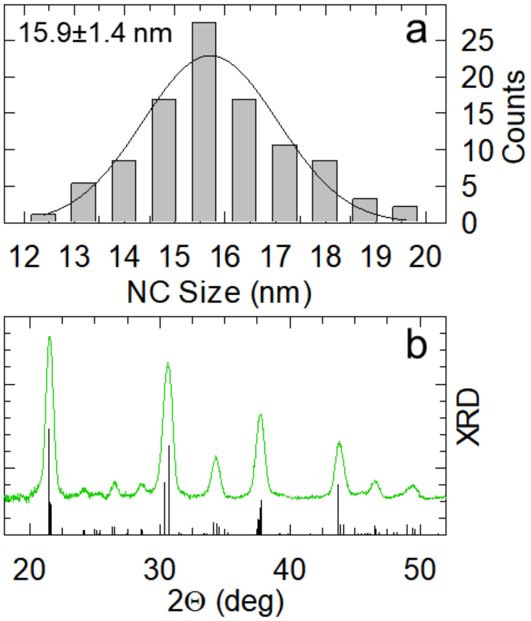

Figure S1: a) $CsPbBr_3$ NCs size distribution extracted from TEM image in Figure 1 showing an average NC size of 15.9 ± 1.4 nm. **b**) X-ray diffractogram of $CsPbBr_3$ NCs together with the reference (ICSD number 97851) pattern of an orthorhombic $CsPbBr_3$ crystal.

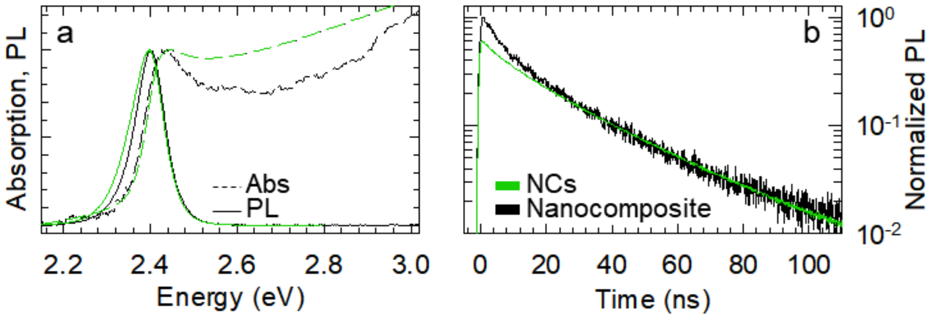

Figure S2: a) Normalized optical absorption (dashed lines) and PL spectra (solid lines) of $CsPbBr_3$ NCs dispersed in colloidal solution (green) and in a 0.1 wt% loaded PLMA nanocomposite. **b)** Tail normalized PL decay traces of the colloidal solution and the nanocomposite. The faster initial component in the nanocomposite reflects the minor optical losses (~20%) introduced by the polymerization process.

Supporting References

1 Imran, M. et al. Shape-Pure, Nearly Monodispersed CsPbBr3 Nanocubes Prepared Using Secondary Aliphatic Amines. Nano Letters 18, 7822-7831, doi:10.1021/acs.nanolett.8b03598 (2018).
